# Wrestling with (in)authenticity

**DOI:** 10.1007/s40037-021-00656-x

**Published:** 2021-03-16

**Authors:** Mario Veen

**Affiliations:** Department of General Practice, Erasmus University Medical Center, Rotterdam, The Netherlands

In their contribution to this issue of *Perspectives on Medical Education*, Désilets et al. [1] connect reflection, Professional Identity Formation (PIF), and authenticity in the context of educational design. They suggest that their educational intervention can contribute to the development of reflection skills, which in turn are conducive to PIF, on the condition that the reflection is authentic: “An important preoccupation when wanting to foster the development of professional identity through the acquisition of reflection skills is the authenticity of students’ reflection. We tried to favor authentic reflection, by having a mentee–mentor pair throughout the entire 4‑year course” [1].

But if the essence of authenticity is its opposition to generalization and instrumentality, isn’t any systematic approach, by definition, inauthentic? Authenticity may be what we all strive for, but the concept seems to contain elements that resist any attempt to apply or harness it. This may be because we are fundamentally unable to distinguish between authenticity and inauthenticity in education through measurement [2]. Or because, in order to *be seen as *authentic, I have to *be *inauthentic [3].

Fortunately, these are the kinds of paradoxes that philosophers love to sink their teeth into. In the following, I summarize some of the travels [4] of authenticity through Western philosophy and explore implications for medical education. I will attempt to answer the question as to what role, if any, authenticity might play in medical education. Or if we should follow Wittgenstein’s advice that “what one cannot speak about must be passed over in silence” [5].

The concept of authenticity has been with us since the birth of Western philosophy. Socrates and Plato wondered how we might distinguish authentic beings from mere shadows [6]. Historically, authenticity had to do with the intentions behind actions and the motivations for living up to ideals, leaving norms and values unquestioned. In a way, this aligns with how the concept is approached in medical education today and brings up another paradox for making authenticity a demand. We encourage our students to take the lead and be autonomous, but only so long as they continue to live up to the standards that we set for them. We require our students to reflect specifically *not* because we require it, but rather because they are intrinsically motivated: do what I say, but not because I say it.

This might be a good place to stress that I do not consider inauthenticity to be intrinsically bad. Indeed, I consider inauthenticity the default. It is both the gateway to authenticity and, in many cases, we simply do not care whether someone is authentic. Whether they perform with sincerity is inconsequential, as long as they behave “competently” and “professionally”. In this sense, we could wonder if we should require authenticity at all. Who cares if a trainee is authentic, as long as they are skilled?

With 19th- and 20th-century existentialist philosophers, authenticity became an explicit focus—perhaps even the core concept. For both Kierkegaard and Nietzsche, it has to do with joyful acceptance of anything that life throws at us [7]. The striving for authenticity is what we all share, but also that which we cannot share. For Kierkegaard, authenticity has to do with my relationship to myself or, if religious, my relationship to God. Much of Nietzsche’s work aimed to rid authenticity of moral connotations and to reject the herd mentality [8]. Authenticity is about acting in a way that expresses who *I* am. In line with how we conceptualize PIF in our field, for Nietzsche, identity is always incomplete and unfinished, and authenticity relates to the degree to which I become who I am, with an emphasis on *becoming*. Authenticity has to do with how I respond to each situation, in each moment, over and over again. In the same conversation with a patient, I may be authentic in one moment (for instance, confessing a personal weakness to a patient instead of trying to embody the all-knowing physician), and inauthentic in the next (being proud of having done so—thus using the patient’s response to achieve an ulterior goal).

So far in our story, there is little hope for authenticity in medical education. It may be too private, too fleeting, and too slippery for us to grasp. But Heidegger, perhaps the philosopher who most explicitly addressed authenticity, built on the idea that authenticity concerns a constant becoming [9]. Like Nietzsche, Heidegger conceptualizes authenticity as an existential assignment for human being that has to be wrested each moment anew—by facing our own existence without worrying what others think of us, or even what we think of ourselves. Yet, for Heidegger, inauthenticity is not worse than authenticity. It simply captures the situation in which we are born into this world and in which we mostly remain. We inherit norms and values, for instance in the form of expectations that our parents have of us, which lead us to pursue a career in medicine and enroll in medical school, or in the form of the curriculum and the demands that medical school places upon us.

The second development made by Heidegger involved a return to the ancient Greek philosophers. Here authenticity became a question of being, and not so much of action or possession. For Heidegger, inauthenticity refers to doing and being as *one* does, and is. ‘One’ here refers to an indeterminate, faceless person in the crowd who does things as ‘people’ do them, as ‘one’ does them, as ‘a physician’ (instead of *this *physician, who *I* am) does them. We could think of an eager first-year student who tries to speak, dress, and act according to their image of ‘how one speaks, dresses, and acts as a physician’.

Authenticity is not the antithesis of inauthenticity, but rather is the way in which we face our inherent inauthenticity. My PIF can be seen as a development from mostly performing physician-related activities as *one* is supposed to perform them—to performing them as *I* (and no one else) performs them. It has to do with embodying who I am as a physician. However, authenticity requires that we courageously confront the existential reality that time is always limited, there is nothing or no one who can tell me how to act in each situation, and that I have to make resolute decisions, sometimes with grave consequences, without ever having all the information to rationally decide the best course of action. For Heidegger, those situations in which I come to face my own existence without any further recourse are the only situations in which we can be truly authentic, until inevitably returning to inauthenticity again. In short, authenticity is not an isolated trait or quality, but the way* I* am in the face of a particular situation. Who I am determines what I do and what skills I acquire, and not the other way around. The idea that I first need to acquire knowledge, skills and attitudes in order to be able to perform professional activities, so that I may ultimately *be *a physician, would for Heidegger be the very definition of inauthenticity.

An essential trait of authenticity is that it does not “look like” anything, by which I mean that there is no specific behavior, way of thinking, attitude, or anything else that we could use to say “*this *is authenticity”. Any example, or indeed any model of reflection, or path to professional identity development, is by definition inauthentic if it becomes a standard for authenticity. Even if Heidegger’s work can be seen as a call to “become who you are”, and we might adopt this in medical education to encourage medical students to “become the physician that you are”, we immediately have to add “… and we cannot give you any guidelines for doing so, nor can we ever know if you succeed”.

With this line of thought, we have returned to a question that Désilets et al. raise. Perhaps we should not concern ourselves with how we can favor authentic reflection, but rather how we—medical students *and *educators—can reflect on authenticity in any meaningful way, so as to contribute to PIF. Sartre and De Beauvoir have worked out implications of Heidegger’s approach to authenticity that may be helpful here.

Sartre describes inauthenticity as a person not embodying or “owning” the situation they are in [10]. He describes a waiter on a terrace who has the performance and attitude of a waiter, without *being *a waiter. The person does the things waiters do, dresses the way they do, and says what waiters tend to say, but his gestures are just a little too articulate. Sartre is quick to stress that this is not a moral judgement on the person in question, but an encouragement for each of us to reflect on our own inauthenticities. In fact, we might draw a parallel with our requirement for medical students to say things like “I’m sorry to hear that” to enact empathy, which can lead to “empathic dissonance”: “the discomfort students experience when pressurised into making empathic statements they don’t sincerely feel” [11, 12]. If we, as medical educators, have the feeling that medical students are acting in ways that seem inauthentic to us, we might do well to wonder who gave them the idea that they should act that way in the first place. Students retreat into mimicking the idea of how ‘one’ does empathy or reflection if they are not encouraged to realize that no one can tell them how to be empathic, or how to reflect. They are free (or perhaps condemned [13]) to invent it in each moment anew.

De Beauvoir develops the notion of “being serious”, describing it in developmental stages that might be used to inform our concept of PIF [14]. For a child (or novice), adults present unfailing examples to live up to—their rules and guidelines solid as rock. A serious person remains in that state of consciousness all their lives and pursues goals and identities as if they can simply be possessed. The maturity of a person (or degree of PIF) is determined by the extent to which they realize that what they had regarded as their role models are also human beings who are, just like them, faced with an ambiguous world in which (unfortunately) no one can tell us how to be, even as a professional. Authenticity is the degree to which I have the courage to embrace this ambiguity, rather than to regard it as a nuisance. Just like Heidegger, we can see that for De Beauvoir, authenticity is not the antithesis of inauthenticity, but rather the way one relates to the brutal fact of the inauthentic default. If inauthenticity has to do with the reification of ideals, we should avoid reifying authenticity for medical students.

But how do we do that? Someone who is inauthentic sees norms and values as facts or things, while someone who is authentic is aware that norms and values are human constructions. I realize that I, and no one else, is responsible both for choosing *these* particular norms and values to adhere to, and for the unique manner in which I interpret and translate general guidelines in concrete situations. A medical student, for instance, is not responsible for the specific guidelines that their educational institution has set for them, but they are responsible for choosing to enroll in an educational institution that sets these guidelines, and for choosing to be trained as a physician in the first place. Authenticity does not mean rejecting all authority, as Nietzsche suggests. Authenticity may, in many cases, require willful submission to authority, especially in the process of becoming part of a community of skilled practitioners [15].

But what does that look like? Authenticity does not “look like” anything. It does not pertain to behavior, attitudes, or skills, but only to the way in which *I* relate to myself. If I have the impression that a medical student is ruthlessly honest to themselves in a portfolio or in a dialogue with me, I might have the impression that they are authentic. They may very well be, but how do I know that their ruthless honesty is not an expression of a tendency to punish themselves, to put themselves down, to only look at their imperfections, and never acknowledge success? Again, this is the trouble we will always run into if we attempt to define authenticity. I could now say that someone who owns their mistakes without passing judgement on them is authentic. However, I would always have to add something like “… as long as this has not become a habit or trick”—i.e., if they do this authentically—which is, of course, begging the question.

Having the same mentor throughout an entire 4‑year course may very well foster authenticity for some students. For others, it might not. This has nothing to do with Désilets et al.’s specific suggestion, but with the nature of authenticity. Does this mean that we have to conclude that authenticity is “useless” for medical education, even if it is perhaps very helpful for medical students? Perhaps, but what Heidegger wrote about philosophy could also apply to authenticity: “It is absolutely correct and proper to say that ‘You can’t do anything with philosophy.’ [But granted] that we cannot do anything with philosophy, might not philosophy, if we concern ourselves with it, do something with us?” [16].

While we cannot do anything with authenticity, we can definitely do something with *in*authenticity in a way that authenticity, in turn, may do something with us. I suggest we apply negative dialectics in a way analogous to Rumi’s often-quoted verse: “Your task is not to seek for love, but merely to seek and find all the barriers within yourself that you have built against it” [17]. The path to authenticity is to reflect on the ways in which I am always already in some way inauthentic.
